# Epithelial Thickness More Than 10 Years After Myopic Laser-Assisted In Situ Keratomileusis (LASIK) Compared With Matched Controls and Its Correlation With Preoperative Refractive Parameters

**DOI:** 10.7759/cureus.98250

**Published:** 2025-12-01

**Authors:** Manogna Nuthi, Mina M Sitto, David G Melanson, Kayvon A Moin, Phillip C Hoopes, Majid Moshirfar

**Affiliations:** 1 College of Osteopathic Medicine, Midwestern University Arizona College of Osteopathic Medicine, Glendale, USA; 2 Hoopes Moshirfar Research Center, Hoopes Vision, Draper, USA; 3 Ophthalmology, Wayne State University School of Medicine, Detroit, USA; 4 Ophthalmology, Rocky Vista University College of Osteopathic Medicine, Ivins, USA; 5 Ophthalmology, Nassau University Medical Center, East Meadow, USA; 6 John A. Moran Eye Center, University of Utah School of Medicine, Salt Lake City, USA; 7 Eye Banking and Corneal Transplantation, Utah Lions Eye Bank, Murray, USA

**Keywords:** corneal epithelial thickness, epithelial remodeling, keratometry, lasik, myopia, myopic regression, pachymetry, sd-oct, visumax, wavelight

## Abstract

Background

This study aimed to investigate corneal epithelial thickness (ET) in patients at least 10 years after laser-assisted in situ keratomileusis (LASIK) compared with a matched control group, using spectral-domain optical coherence tomography (SD-OCT).

Methodology

This retrospective study analyzed 125 eyes (68 patients) who underwent myopic LASIK between 1996 and 2014 and compared them with a control group of 150 eyes (76 patients) with no prior ocular surgery. The control group was matched to the LASIK cohort based on age, sex, postoperative sphere, and postoperative spherical equivalent (SEQ), and included only eyes with available OCT imaging and no history of ocular pathology or surgery. In addition, correlations were evaluated between preoperative sphere, SEQ, mean keratometry, and postoperative ET maps. Corneal pachymetry was measured at the 2-, 5-, 7-, and 9-mm optical zone diameters using the Avanti Widefield OCT (Optovue, Fremont, CA, USA). Corneal topography metrics such as central corneal thickness and keratometry were obtained using Pentacam HR (Oculus, Wetzlar, Germany).

Results

The LASIK group had greater ET by 2.3 ± 0.5 µm in the central 2-mm zone compared with the matched control group (p < 0.001), with consistently thicker epithelium across the 5-mm zone. In contrast, ET was significantly thinner in the nasal and inferior regions of the 7- and 9-mm zones, as well as the temporal region of the 9-mm zone (all p < 0.05). ET variability, maximum ET, and minimum-maximum ET were increased following LASIK compared with control (p < 0.001), whereas minimum ET was lower by 2.6 ± 0.6 µm (p < 0.001). Correlation analysis showed that both preoperative sphere and SEQ were positively correlated with ET in the 7- and 9-mm zones (all p < 0.05), whereas no significant correlations were observed in the central or paracentral regions. Preoperative central corneal thickness showed a modest negative correlation with ET in the 2- and 5-mm zones, the nasal and inferior-nasal regions of the 7- and 9-mm zones, and the temporal region of the 9-mm zone (p < 0.05).

Conclusions

Long-term follow-up of LASIK patients revealed sustained central and paracentral epithelial thickening, indicating that epithelial remodeling persists beyond the short- and mid-term periods documented in the literature. While the central and paracentral cornea remained thicker after LASIK than control, the periphery was thinner, predominantly in the nasal and inferior regions and extending to the temporal region at the 9-mm zone. Higher preoperative myopia was associated with thinner epithelium in regions of the peripheral cornea. In addition, thinner preoperative central corneas demonstrated greater epithelial thickening in the central and paracentral zones, suggesting that baseline corneal thickness may impact the magnitude of epithelial remodeling. The epithelial distribution pattern after myopic LASIK is non-uniform and may contribute to long-term refractive changes.

## Introduction

Laser-assisted in situ keratomileusis (LASIK) was first introduced in 1989 by Dr. Gholam Peyman to correct refractive error by targeting the cornea. It has since become a common ophthalmologic surgery performed worldwide, with nearly one million patients undergoing LASIK each year in the United States [1,2]. LASIK primarily acts on the most anterior cornea, while the mid- and posterior stroma, Descemet’s membrane, and corneal endothelium are preserved [3]. Corneal wound healing following stromal ablation involving epithelial hyperplasia and stromal remodeling is critical to postoperative visual quality [4]. The corneal epithelium, despite a central thickness of approximately 50 to 52 µm, contributes a mean of 1.03 diopters (D) to the central corneal power [5,6]. Its measurable impact on corneal refraction and its tendency to remodel following LASIK have shown important implications for long-term refractive stability [4,7]. Understanding these remodeling patterns may help improve predictability following LASIK.

Furthermore, spectral-domain optical coherence tomography (SD-OCT) has been shown to be a valuable tool for assessing central epithelial thickness (ET), allowing precise measurement and identification of areas with abnormal thickness [8]. Following a corneal refractive procedure, the epithelium typically renews through peripheral epithelial cell proliferation and centripetal migration of epithelial stem cells from the limbus, mediated by epidermal growth factor induction [9]. This process is accelerated during wound healing, often resulting in a period of epithelial remodeling [4,9]. While some studies have reported that epithelial remodeling contributes to refractive regression, this association remains debated [7,10-12].

Epithelial remodeling patterns have been documented as early as the first month post-surgery, continuing for up to two years, with evidence of changes persisting for up to seven years post-LASIK [7,11-16]. However, to our knowledge, there have been no studies examining ET beyond the seven-year mark. The primary objective of this study was to evaluate ET more than 10 years after LASIK in comparison with a matched control group using SD-OCT measurements.

## Materials and methods

Study design

This retrospective study included 68 de-identified patients (125 eyes) who underwent LASIK between 1996 and 2014. Data were collected from a single refractive surgery center in Draper, Utah. All participants provided written consent at the time of their initial evaluation for the anonymized use of their data, in accordance with the Declaration of Helsinki, and full approval was granted by the Biomedical Research Alliance of New York (approval number: A20-12-547-823).

The inclusion criteria required patients to have undergone myopic LASIK in one or both eyes and follow-up OCT measurements of the 2-mm to 9-mm optical zone diameters available at least 10 years after surgery. Exclusion criteria included patients who had other ophthalmic procedures (e.g., enhancements, hyperopic LASIK, cataract surgery, corneal cross-linking, retinal surgery, or epithelial scraping), as well as those with corneal and/or retinal abnormalities. A control group of 150 eyes (76 patients) with no history of ocular surgery was randomly selected from an anonymized list of patients with recent OCT imaging and matched to the LASIK group based on age, sex, postoperative sphere, and postoperative manifest refraction spherical equivalent (SEQ). Exclusion criteria for this group included any history of ophthalmic surgery, ocular pathology, or abnormal topography.

All patients underwent ophthalmic evaluations at baseline and at follow-up visits, with at least 10 years of follow-up, including slit-lamp biomicroscopy examination, SEQ, uncorrected distance visual acuity, corrected distance visual acuity, keratometry, and pachymetry. Central corneal thickness (CCT) and mean anterior keratometry (Km) values were obtained using the Pentacam HR (Oculus, Wetzlar, Germany) within a 6-mm zone centered at the corneal vertex. Corneal pachymetry within the 7-mm zone (17 areas) was measured using the Avanti Widefield OCT (Optovue, Fremont, CA, USA; software version 2018.1.0.43). ET was assessed centrally at a 2-mm diameter and in eight octants (superior, superior-nasal, nasal, inferior-nasal, inferior, inferior-temporal, temporal, and superior-temporal) within the 5-, 7-, and 9-mm zones. In addition, maximum ET, minimum ET, thickness variation (minimum-maximum), and ET variability (standard deviation across 17 imaged areas) were obtained from the OCT.

Surgical intervention

All LASIK procedures were performed by a single surgeon (MM) at the same location. The femtosecond-assisted laser was used to create a superiorly hinged flap with an 8.5- to 9.0-mm diameter and 100- to 110-μm thickness. Following flap creation, laser stromal ablation was performed with a 6.0- to 6.5-mm central optical zone and an 8.5- to 9.0-mm transition zone. The eye was subsequently irrigated, and the corneal flap was repositioned. Postoperative management included moxifloxacin 0.5% (Alcon Laboratories, Inc., Fort Worth, TX, USA) four times per day for one week and prednisolone acetate 1% (Falcon Pharmaceuticals Ltd., Fort Worth, TX, USA) every hour while awake for the first day, then four times per day for one week.

Statistical analysis

Statistical analyses were conducted using SPSS version 29 (IBM Corp., Armonk, NY, USA). Normality of the data was evaluated with the Shapiro-Wilk test. Demographic variables (age and sex) were analyzed at the patient level using independent samples t-tests for continuous data and chi-square tests for categorical data. A generalized estimating equations (GEE) regression model with exchangeable correlation structure was applied using Python (version 3.13; Python Software Foundation, Fredericksburg, VA, USA) to account for intereye correlation. Standardized β coefficients (r) derived from GEE linear regression models were calculated to assess associations between preoperative characteristics and postoperative ET variables. A p-value of less than 0.05 was considered statistically significant.

## Results

Preoperative characteristics

A total of 125 eyes (68 patients) underwent LASIK and were compared to a matched control group of 150 eyes (76 patients) (Table 1). The mean follow-up time after LASIK was 17 ± 5 years (range = 10 to 27). The LASIK group had a mean age of 54 ± 11 years (range = 36 to 78), comprising 30 (43.2%) females and 38 (56.8%) males. In comparison, the control group had a mean age of 53 ± 12 years (range = 35 to 76) with 33 (43.3%) females and 43 (56.7%) males. Post-LASIK sphere and SEQ did not differ statistically from the control group (p > 0.05), whereas Km and CCT differed significantly from those of control eyes (both p < 0.001).

**Table 1:** Patient characteristics. Values are expressed as mean ± standard deviation (range) unless otherwise noted. *: Indicates statistically significant difference (p < 0.05). ^a^: Statistical differences between the control and postoperative LASIK groups were determined using independent samples t-tests, chi-square tests, or generalized estimating equations. LASIK = laser-assisted in situ keratomileusis; D = diopters; SEQ = spherical equivalent; Km = mean anterior keratometry; CCT = central corneal thickness

Parameter	Preoperative LASIK	Postoperative LASIK	Control	P-value^a^	t/*χ*² value
	n = 68	n = 68	n = 76		
Age (years)	38 ± 11 (20 to 64)	54 ± 11 (36 to 78)	53 ± 12 (35 to 76)	0.68	0.51
Female/Male (n, %)	30 (43.2%) / 38 (56.8%)	30 (43.2%) / 38 (56.8%)	33 (43.3%) / 43 (56.7%)	0.94	0.08
	n = 125	n = 125	n = 150		
Sphere (D)	-3.66 ± 2.29 (-9.75 to -0.25)	-0.87 ± 0.48 (-1.75 to -0.25)	-0.81 ± 0.70 (-2.2 to 0.60)	0.40	0.85
SEQ (D)	-4.00 ± 2.38 (-10.00 to -0.38)	-0.68 ± 0.51 (-1.25 to 0.50)	-0.71 ± 0.48 (-1.20 to 0.10)	0.60	0.53
Km (D)	43.87 ± 1.58 (39.75 to 46.90)	39.37 ± 1.80 (35.78 to 41.13)	42.64 ± 1.75 (41.80 to 46.22)	<0.001*	11.03
CCT (µm)	557 ± 28 (506 to 614)	497 ± 32 (431 to 511)	531 ± 27 (493 to 587)	<0.001*	6.85

Corneal epithelial map analysis

At the most recent follow-up (10 years or longer), the mean central ET for the LASIK group was 2.3 ± 0.5 µm thicker than that of the control group (56.6 ± 4.3 vs. 54.3 ± 3.6 µm, respectively; p < 0.001) (Table 2). In the 5-mm area, the difference between superior and inferior regions did not statistically differ between LASIK and control eyes, indicating that the inferior section was thicker than the superior (p = 0.796) (Table 3). Additionally, the LASIK group consistently had thicker epithelium across all regions within the 5-mm zone in comparison to the control (p < 0.05) (Table 2).

**Table 2:** Epithelial thickness analysis between LASIK and control. Values are expressed as mean ± standard deviation. *: Indicates statistically significant difference using generalized estimating equations (p < 0.05). LASIK = laser-assisted in situ keratomileusis; ET = epithelial thickness

Parameter (µm)	LASIK (n = 125)	Control (n = 150)	P-value
2-mm central ET	56.6 ± 4.3	54.3 ± 3.6	<0.001*
5-mm zone			
Superior ET	54.7 ± 4.5	52.9 ± 3.6	0.002*
Superior-nasal ET	55.4 ± 4.0	53.7 ± 3.7	0.003*
Nasal ET	56.3 ± 3.9	54.4 ± 3.6	<0.001*
Inferior-nasal ET	56.7 ± 4.1	55.0 ± 3.6	<0.001*
Inferior ET	57.0 ± 4.4	55.2 ± 3.8	0.003*
Inferior-temporal ET	56.8 ± 4.3	54.3 ± 3.9	<0.001*
Temporal ET	56.4 ± 4.4	53.1 ± 3.8	<0.001*
Superior-temporal ET	55.1 ± 4.5	52.6 ± 3.6	<0.001*
7-mm zone			
Superior ET	50.9 ± 4.7	51.2 ± 4.0	0.634
Superior-nasal ET	52.1 ± 4.2	53.2 ± 3.5	0.045*
Nasal ET	52.8 ± 4.2	54.0 ± 3.5	0.018*
Inferior-nasal ET	53.0 ± 4.2	54.4 ± 3.4	0.015*
Inferior ET	53.3 ± 4.6	54.7 ± 3.8	0.014*
Inferior-temporal ET	53.3 ± 4.2	54.3 ± 3.6	0.083
Temporal ET	52.2 ± 4.2	52.3 ± 3.5	0.322
Superior-temporal ET	50.7 ± 4.2	51.0 ± 3.6	0.544
9-mm zone			
Superior ET	48.2 ± 4.5	48.8 ± 5.2	0.464
Superior-nasal ET	51.1 ± 4.6	52.1 ± 4.4	0.123
Nasal ET	52.5 ± 4.2	54.4 ± 4.1	0.002*
Inferior-nasal ET	51.4 ± 4.2	53.5 ± 3.9	<0.001*
Inferior ET	49.6 ± 4.9	52.9 ± 4.1	<0.001*
Inferior-temporal ET	50.0 ± 4.1	53.3 ± 3.3	<0.001*
Temporal ET	48.7 ± 4.1	51.0 ± 3.7	<0.001*
Superior-temporal ET	47.7 ± 4.0	48.9 ± 4.4	0.058

**Table 3:** Superior-inferior corneal epithelial thickness differences across 5-, 7-, and 9-mm zones in LASIK versus control. Values are expressed as mean ± standard deviation. *: Indicates statistically significant difference using generalized estimating equations (p < 0.05). LASIK = laser-assisted in situ keratomileusis

Superior-inferior mean difference (µm)	LASIK (n = 125)	Control (n = 150)	P-value
5-mm zone	-2.3 ± 3.3	-2.3 ± 2.6	0.796
7-mm zone	-2.4 ± 4.5	-3.4 ± 3.8	0.064
9-mm zone	-1.3 ± 5.4	-4.1 ± 5.2	<0.001*

**Figure 1:**
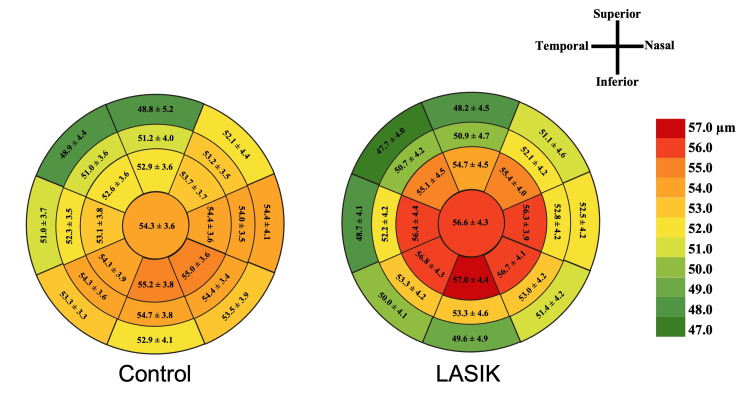
Comparison of epithelial thickness maps between LASIK and control. Values represent mean ± standard deviation across each optical zone. Color coding corresponds to epithelial thickness based on the scale shown, with warmer colors indicating thicker epithelium and cooler colors indicating thinner regions. LASIK = laser-assisted in situ keratomileusis

The LASIK group had thinner ET measurements along the superior-nasal (p = 0.045), nasal (p = 0.018), inferior-nasal (p = 0.015), and inferior (p = 0.014) regions of the 7-mm zone compared with control eyes (Table 2 and Figure 1). Similarly, the 9-mm peripheral zone also showed thinner ET values along those regions, including the inferior-temporal and temporal regions (all p < 0.001). The control group showed a greater mean difference between the superior and inferior regions compared to the LASIK group for the 9-mm zone (mean difference = -4.1 ± 5.2 vs -1.3 ± 5.4 µm; p < 0.001) and the 7-mm zone (mean difference = -3.4 ± 3.8 vs -2.4 ± 4.5 µm), though the latter was not statistically significant (p = 0.064) (Table 3).

**Figure 2:**
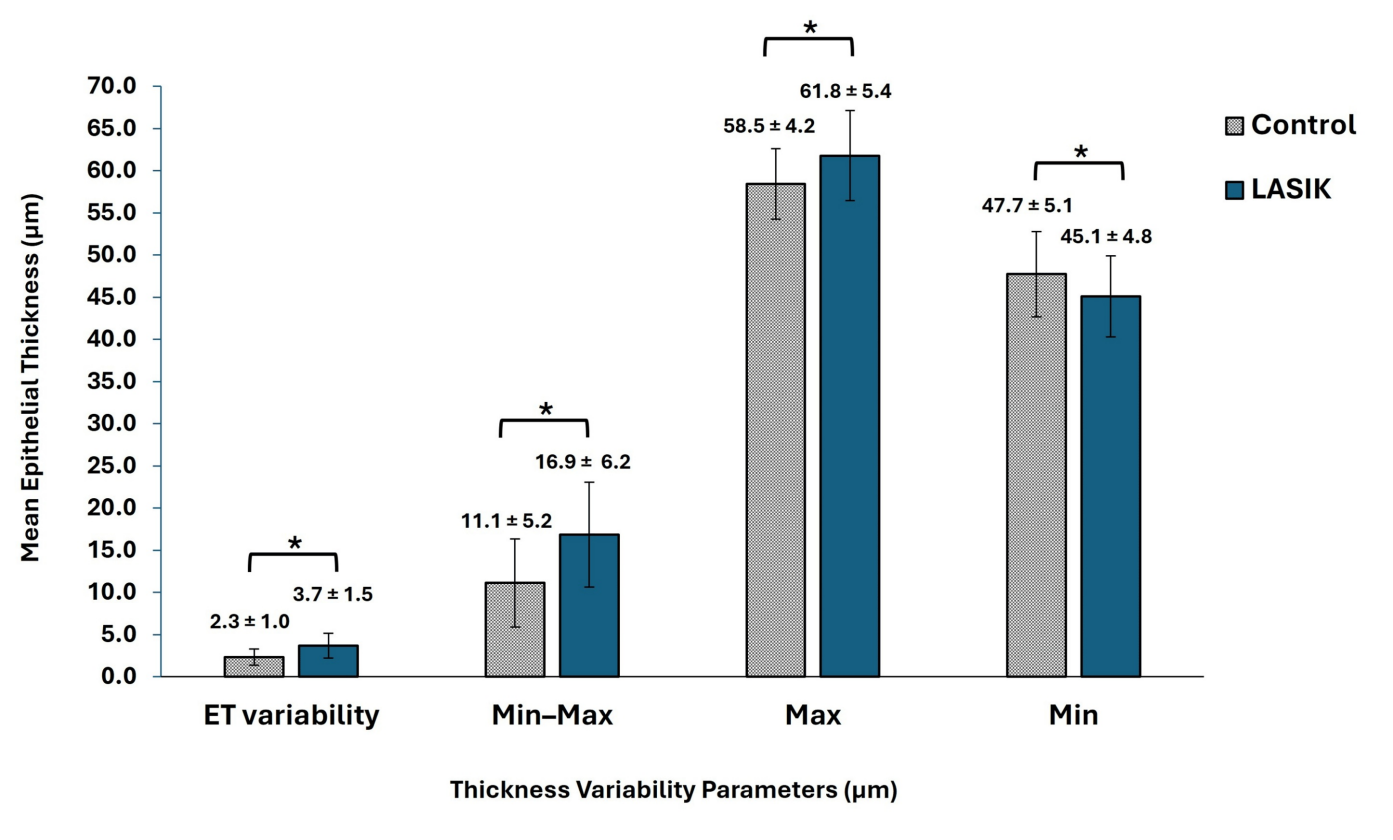
Comparison of epithelial thickness variables between LASIK and control. Values are expressed as mean ± standard deviation. *: Indicates statistically significant difference (p < 0.05). LASIK = laser-assisted in situ keratomileusis; ET = epithelial thickness; Max = maximum; Min = minimum

There were significant differences across all four parameters between the LASIK and control groups (Figure 2). Specifically, the mean ET variability was greater following LASIK than in the control group by 1.5 µm (3.7 ± 1.5 vs. 2.3 ± 1.0 µm; p < 0.001). The minimum-maximum and maximum ET values were also greater in the LASIK group compared with the control group by 5.8 ± 0.8 µm (16.9 ± 6.2 vs. 11.1 ± 5.2 µm; p < 0.001) and 3.3 ± 0.6 µm (61.8 ± 5.4 vs. 58.5 ± 4.2 µm; p < 0.001), respectively. In contrast, the mean minimum ET value was lower for LASIK in comparison to control by 2.6 ± 0.6 µm (45.1 ± 4.8 vs. 47.7 ± 5.1 µm; p < 0.001). All these parameters indicate increased variability in the ET profile after LASIK.

Correlations between preoperative characteristics and epithelial thickness zones

There was a negative correlation between preoperative sphere and postoperative ET in the 2-mm zone, although not statistically significant (r = -0.498; p = 0.121) (Table 4). These findings were consistent in the 5-mm zone (all p > 0.05). The 7-mm zone, however, showed positive correlations in all regions (p < 0.05), except for the superior and temporal regions (p > 0.05). The 9-mm zone also showed positive correlations in the nasal, inferior-nasal, inferior, inferior-temporal, and temporal regions (all p < 0.05). SEQ demonstrated a correlation pattern similar to that of the sphere across all zones and regions. ET variability and minimum-maximum showed moderate negative correlations with both sphere (r = -0.398 and r = -0.839; p < 0.001 and p = 0.017, respectively) and SEQ (r = -0.356 and r = -0.762; p = 0.002 and p = 0.046, respectively) (Table 5).

**Table 4:** Correlation analysis of preoperative visual parameters and long-term corneal epithelial thickness across 2-, 5-, 7-, and 9-mm zones after LASIK. Values expressed as standardized β coefficients, r (p-value). Bolded values indicate statistically significant difference using generalized estimating equations (p < 0.05). SEQ = spherical equivalent; Km = mean anterior keratometry; CCT = central corneal thickness

Parameter (µm)	Sphere	SEQ	Km	CCT
2-mm central ET	-0.498 (0.121)	-0.514 (0.109)	0.480 (0.164)	-0.060 (0.010)
5-mm zone				
Superior ET	-0.120 (0.697)	-0.174 (0.506)	0.077 (0.817)	-0.056 (0.039)
Superior-nasal ET	-0.097 (0.727)	-0.147 (0.559)	0.153 (0.597)	-0.050 (0.046)
Nasal ET	-0.290 (0.329)	-0.337 (0.235)	0.657 (0.137)	-0.056 (0.006)
Inferior-nasal ET	-0.340 (0.283)	-0.352 (0.290)	0.806 (0.037)	-0.061 (0.003)
Inferior ET	-0.430 (0.207)	-0.370 (0.326)	0.843 (0.022)	-0.041 (0.074)
Inferior-temporal ET	-0.407 (0.270)	-0.397 (0.314)	0.465 (0.265)	-0.062 (0.010)
Temporal ET	-0.504 (0.135)	-0.504 (0.147)	0.264 (0.481)	-0.066 (0.015)
Superior-temporal ET	-0.290 (0.365)	-0.270 (0.386)	0.135 (0.701)	-0.065 (0.030)
7-mm zone				
Superior ET	0.490 (0.051)	0.448 (0.065)	0.319 (0.334)	0.076 (0.52)
Superior-nasal ET	0.520 (0.043)	0.486 (0.033)	-0.021 (0.735)	-0.045 (0.051)
Nasal ET	0.743 (<0.001)	0.678 (<0.001)	0.497 (0.123)	-0.055 (0.003)
Inferior-nasal ET	0.697 (<0.001)	0.638 (<0.001)	0.581 (0.100)	-0.058 (<0.001)
Inferior ET	0.602 (0.003)	0.578 (<0.001)	0.640 (0.173)	-0.032 (0.107)
Inferior-temporal ET	0.711 (<0.001)	0.696 (<0.001)	0.316 (0.467)	-0.031 (0.060)
Temporal ET	0.132 (0.26)	0.143 (0.21)	0.081 (0.350)	0.049 (0.443)
Superior-temporal ET	0.752 (0.003)	0.716 (0.003)	-0.125 (0.715)	-0.031 (0.243)
9-mm zone				
Superior ET	0.052 (0.883)	-0.005 (0.90)	0.359 (0.363)	-0.009 (0.892)
Superior-nasal ET	0.311 (0.467)	0.239 (0.560)	-0.373 (0.357)	-0.061 (0.294)
Nasal ET	0.627 (0.009)	0.588 (0.009)	0.070 (0.843)	-0.046 (0.019)
Inferior-nasal ET	0.551 (0.016)	0.520 (0.016)	0.163 (0.621)	-0.048 (0.026)
Inferior ET	0.604 (0.003)	0.514 (0.014)	0.668 (0.225)	-0.033 (0.416)
Inferior-temporal ET	0.825 (<0.001)	0.767 (<0.001)	0.477 (0.253)	-0.054 (0.124)
Temporal ET	0.813 (<0.001)	0.762 (<0.001)	0.486 (0.104)	-0.054 (0.044)
Superior-temporal ET	0.274 (0.455)	0.245 (0.500)	0.033 (0.919)	-0.037 (0.437)

**Table 5:** Correlation of preoperative visual parameters with long-term ET variability, maximum, minimum, and thickness variation (Min–Max) after LASIK. Values expressed as standardized β coefficients, r (p-value). Bolded values indicate statistically significant difference using generalized estimating equations (p < 0.05). SEQ = spherical equivalent; Km = mean anterior keratometry; CCT = central corneal thickness; ET = epithelial thickness; Min = minimum; Max = maximum

Parameter (µm)	Sphere	SEQ	Km	CCT
ET variability	-0.398 (<0.001)	-0.356 (0.002)	0.048 (0.688)	-0.010 (0.203)
Min–max ET	-0.839 (0.017)	-0.762 (0.046)	0.431 (0.326)	-0.043 (0.140)
Max ET	-0.498 (0.182)	-0.528 (0.157)	0.644 (0.126)	-0.063 (0.034)
Min ET	0.442 (0.106)	0.400 (0.141)	-0.071 (0.800)	-0.012 (0.525)

In the 5-mm zone, there was a positive correlation between preoperative Km and the inferior-nasal (r = 0.806; p = 0.03) and inferior ET regions (r = 0.843; p = 0.022) (Table 4). All other zones showed no statistically significant difference (p > 0.05). Regarding preoperative CCT, there was an inverse correlation with ET in the 2-mm zone (r = -0.060; p = 0.010) and across all regions in the 5-mm zone (p < 0.05), except in the inferior region (p > 0.05). This was also observed peripherally in the nasal and inferior-nasal regions of the 7-mm zone (r = -0.055 and -0.058; p = 0.003 and p < 0.001, respectively) and the 9-mm zone (r = -0.046 and -0.48; p = 0.019 and p = 0.026, respectively), as well as in the temporal region of the 9-mm zone (r = -0.054; p = 0.044). Maximum ET and preoperative CCT also showed a slight inverse relationship (r = -0.63; p = 0.034).

## Discussion

Epithelial thickness patterns after LASIK represent an important consideration for refractive surgeons, given their influence on both surgical planning and the predictability of postoperative refractive outcomes [12]. Our study evaluated long-term corneal epithelial remodeling following LASIK, with follow-up data extending beyond 10 years. Previous studies have primarily investigated short-term (one to two years) or mid-term (up to seven years) outcomes, with limited long-term data [7,11-16]. Our findings demonstrate a persistent elevation in ET among LASIK patients compared with the control group, particularly in the 2- and 5-mm optical zones. Furthermore, the greater maximum ET and reduction in minimum ET after LASIK indicate sustained epithelial hyperplasia. One study reported a persistent increase in minimum-maximum over a two-year period following LASIK [7]. Patel et al. demonstrated that ET remained elevated at seven years at a mean of 52 ± 6 µm, similar to our group at 10 years and greater [11]. Moreover, the pattern of thickening seen in our study is consistent with the lenticular pattern described by Reinstein et al., who explained that central thickening may serve as a compensatory response to stromal thinning [14]. Reinstein et al. reported central and paracentral thickening during the first one to three months post-LASIK, with increases of 1.7 µm at the corneal vertex, 1 µm at the 2-mm radius, and 0.3 µm at the 3-mm radius [14]. By one year, however, all eyes (n = 10) demonstrated paracentral epithelial thinning within the annulus between the 3- to 3.4-mm radius from the corneal vertex, consistent with our findings in the 7- to 9-mm diameter areas [14].

OCT imaging technology primarily measures over a 6-mm corneal diameter, which is smaller than the 6.5-mm optical zone in LASIK [17]. In contrast, SD-OCT captures a 9-mm diameter, which provides a wider assessment of the ET profile [17]. Using SD-OCT, we observed that epithelial map analysis more than 10 years after LASIK demonstrated distinct regional patterns in ET across the cornea. The central and paracentral areas were significantly thicker after LASIK than the control, while the periphery showed thinning, predominantly in the nasal and inferior regions and extending to the temporal region at the 9-mm zone. This peripheral thinning is consistent with reported literature describing a centrifugal ET gradient with a decrease of -0.43 µm/mm toward the 7-mm zone and a steeper decrease of -2.31 µm/mm toward the 9-mm zone [18]. The mean ET maps also showed thinner corneal epithelium in superior regions and thicker epithelium in the inferior regions, although significantly only in the peripheral 9-mm zone. This asymmetry has been observed as early as three months and as late as five years postoperatively [19,20], which suggests that LASIK induces a regionally non-uniform pattern of epithelial distribution.

Our correlation data showed that higher preoperative myopia was associated with thinner ET in the peripheral cornea (7-mm and 9-mm zones), particularly in the nasal and inferior regions. Several studies have demonstrated a correlation between the degree of myopia correction and ET thickening, although findings have been inconsistent [10,11,15]. Kanellopoulos et al. reported an increase of 1 µm per degree of myopic ablation within the 5-mm zone [15]. In contrast, another study reported central epithelial thickening rates of 2.1 μm/D for low myopia (−1.00 and −4.00 D), 1.8 μm/D for moderate myopia (−4.25 and −6.00 D), and 1.5 μm/D for high myopia (−6.25 and −13.50 D) [21]. Although this relationship may appear linear, ET changes may plateau in moderate-to-high myopic corrections due to biological constraints.

Corneal wound healing is thought to contribute to the gradual thickening of the cornea [22]. In our study, we observed relatively weak negative correlations between preoperative CCT and ET within the 2- and 5-mm zones, as well as the nasal regions in the periphery. This may suggest that thinner corneas at baseline undergo greater epithelial thickening after LASIK. One possible explanation is that thin preoperative CCT may lead to weaker postoperative corneal tensile strength due to its effect on biomechanical properties [23]. However, the epithelial response is primarily during the early postoperative period. Zhao et al. observed that early postoperative thickening after thin-flap LASIK in their Chinese population resulted from stromal compensation [22], whereas our data are more consistent with epithelial remodeling as the compensatory mechanism. Our findings support the hypothesis that remodeling occurs to smooth the stromal irregularities created by myopic ablation [24]. This compensatory response results in epithelial thickening within the 5-mm zone and relative thinning in the peripheral 7- to 9-mm zone after myopic refractive surgery [12,24].

When evaluating preoperative keratometry, however, we found no correlation with ET after LASIK, with the exception of a positive correlation in the 5-mm inferior and inferior-nasal regions. These findings are relatively consistent with prior studies showing that keratometry is not correlated with ET in control eyes [18]. Myopic regression, however, is thought to be influenced by the healing response of the cornea [25], and prior studies have reported conflicting associations between myopic regression and corneal curvature. One study identified steep preoperative corneal curvature as a strong predictor of myopic regression at one year [26], while another study found greater regression in flatter corneas after LASIK [24]. Reinstein et al. proposed that larger changes in the corneal curvature gradient are correlated with greater epithelial remodeling, which may increase the risk of myopic regression [24].

This study has several limitations, including the retrospective design and limited availability of preoperative data. Our approach was to select patients in the control group with similar demographics and refractive characteristics to postoperative LASIK, including age, sex, sphere, and SEQ. This helps reduce potential confounding, especially because OCT imaging technology was not available 10 years ago at our refractive surgery center [6]. The inclusion criteria required a follow-up of at least 10 years after LASIK, which introduced variability in time since surgery and may have affected the degree of observed remodeling. We also did not analyze longitudinal trends in ET or refractive changes over time, as this was beyond the scope of our study. Despite these limitations, our findings indicate that corneal epithelial remodeling can persist for at least a decade after LASIK.

## Conclusions

This retrospective study examined ET across a 9-mm diameter in eyes with more than 10 years of follow-up after LASIK. Our findings indicate a sustained increase in ET, particularly in the central and paracentral zones, suggesting ongoing epithelial remodeling well beyond the short- and mid-term periods previously documented in the literature. In addition, higher preoperative myopia was associated with thinner epithelium in the peripheral cornea. Thinner preoperative central corneas showed more central and paracentral epithelial thickening, indicating that baseline corneal thickness may impact the magnitude of epithelial remodeling. These results emphasize the importance of monitoring corneal ET in LASIK patients, as persistent thickening may influence postoperative refractive stability, including a tendency toward myopic regression, as well as visual quality outcomes. Future prospective longitudinal studies with standardized protocols are needed to determine whether preoperative characteristics, such as the level of myopia, can predict the degree of hyperplasia.

## References

[1] Solomon KD, Fernández de Castro LE, Sandoval HP (2009). LASIK world literature review: quality of life and patient satisfaction. Ophthalmology.

[2] Bailey MD, Zadnik K (2007). Outcomes of LASIK for myopia with FDA-approved lasers. Cornea.

[3] McAlinden C (2012). Corneal refractive surgery: past to present. Clin Exp Optom.

[4] Wilson SE, Mohan RR, Hong JW, Lee JS, Choi R, Mohan RR (2001). The wound healing response after laser in situ keratomileusis and photorefractive keratectomy: elusive control of biological variability and effect on custom laser vision correction. Arch Ophthalmol.

[5] Reinstein DZ, Archer TJ, Gobbe M, Silverman RH, Coleman DJ (2008). Epithelial thickness in the normal cornea: three-dimensional display with Artemis very high-frequency digital ultrasound. J Refract Surg.

[6] Fujimoto J, Swanson E (2016). The development, commercialization, and impact of optical coherence tomography. Invest Ophthalmol Vis Sci.

[7] Kanellopoulos AJ (2019). Comparison of corneal epithelial remodeling over 2 years in LASIK versus SMILE: a contralateral eye study. Cornea.

[8] Tañá-Rivero P, Orts-Vila P, Tañá-Sanz P, Ramos-Alzamora M, Montés-Micó R (2024). Assessment of corneal epithelial thickness mapping by spectral-domain optical coherence tomography. Front Med (Lausanne).

[9] Liu CY, Kao WW (2015). Corneal epithelial wound healing. Prog Mol Biol Transl Sci.

[10] Yan MK, Chang JS, Chan TC (2018). Refractive regression after laser in situ keratomileusis. Clin Exp Ophthalmol.

[11] Patel SV, Erie JC, McLaren JW, Bourne WM (2007). Confocal microscopy changes in epithelial and stromal thickness up to 7 years after LASIK and photorefractive keratectomy for myopia. J Refract Surg.

[12] Zhu M, Xin Y, Vinciguerra R (2023). Corneal epithelial remodeling in a 6-month follow-up period in myopic corneal refractive surgeries. J Refract Surg.

[13] Díaz-Bernal J, García-Basterra I, Mora-Castilla J, Nguyen A, Fernandez-Barrientos Y, Guerrero AM (2021). Evolution of corneal epithelial remodeling after myopic laser in situ keratomileusis surgery measured by anterior segment optical coherence tomography combined with Placido disk. Indian J Ophthalmol.

[14] Reinstein DZ, Archer TJ, Gobbe M (2012). Change in epithelial thickness profile 24 hours and longitudinally for 1 year after myopic LASIK: three-dimensional display with Artemis very high-frequency digital ultrasound. J Refract Surg.

[15] Kanellopoulos AJ, Asimellis G (2014). Longitudinal postoperative lasik epithelial thickness profile changes in correlation with degree of myopia correction. J Refract Surg.

[16] Moilanen JA, Holopainen JM, Vesaluoma MH, Tervo TM (2008). Corneal recovery after lasik for high myopia: a 2-year prospective confocal microscopic study. Br J Ophthalmol.

[17] Fan L, Xiong L, Zhang B, Wang Z (2019). Longitudinal and regional non-uniform remodeling of corneal epithelium after topography-guided FS-LASIK. J Refract Surg.

[18] Abtahi MA, Beheshtnejad AH, Latifi G (2024). Corneal epithelial thickness mapping: a major review. J Ophthalmol.

[19] Tang M, Li Y, Huang D (2015). Corneal epithelial remodeling after LASIK measured by Fourier-domain optical coherence tomography. J Ophthalmol.

[20] Li Y, Tan O, Brass R, Weiss JL, Huang D (2012). Corneal epithelial thickness mapping by Fourier-domain optical coherence tomography in normal and keratoconic eyes. Ophthalmology.

[21] Reinstein DZ, Srivannaboon S, Gobbe M, Archer TJ, Silverman RH, Sutton H, Coleman DJ (2009). Epithelial thickness profile changes induced by myopic LASIK as measured by Artemis very high-frequency digital ultrasound. J Refract Surg.

[22] Zhao MH, Wu Q, Jia LL, Hu P (2015). Changes in central corneal thickness and refractive error after thin-flap laser in situ keratomileusis in Chinese eyes. BMC Ophthalmol.

[23] Wu Y, Shen T, Tan L, He T, Zheng Q, Hong C (2023). Corneal remodeling after SMILE for moderate and high myopia: short-term assessment of spatial changes in corneal volume and thickness. BMC Ophthalmol.

[24] Reinstein DZ, Archer TJ, Gobbe M (2014). Rate of change of curvature of the corneal stromal surface drives epithelial compensatory changes and remodeling. J Refract Surg.

[25] Manion GN, Moin KA, Brown AH, Olson TV, Kezirian GM, Hoopes PC, Moshirfar M (2025). Preoperative risk factors of keratometry, myopia, astigmatism, age, and sex for myopic regression after laser-assisted in situ keratomileusis, photorefractive keratectomy, and keratorefractive lenticule extraction. Cornea.

[26] Mohamed Mostafa E (2015). Effect of flat cornea on visual outcome after LASIK. J Ophthalmol.

